# To 1000 Gy and back again: a systematic review on dose-response evaluation in selective internal radiation therapy for primary and secondary liver cancer

**DOI:** 10.1007/s00259-021-05340-0

**Published:** 2021-04-10

**Authors:** Joey Roosen, Nienke J. M. Klaassen, Lovisa E. L. Westlund Gotby, Christiaan G. Overduin, Marcel Verheij, Mark W. Konijnenberg, J. Frank W. Nijsen

**Affiliations:** 1 Department of Medical Imaging, Radboud Institute for Health Sciences, Radboud University Medical Center, Nijmegen, The Netherlands; 2 Department of Radiation Oncology, Radboud University Medical Center, Nijmegen, The Netherlands; 3 Department of Radiology and Nuclear Medicine, Erasmus Medical Center, Rotterdam, The Netherlands

**Keywords:** SIRT, Radioembolization, TARE, Yttrium, Holmium, Dosimetry

## Abstract

**Purpose:**

To systematically review all current evidence into the dose-response relation of yttrium-90 and holmium-166 selective internal radiation therapy (SIRT) in primary and secondary liver cancer.

**Methods:**

A standardized search was performed in PubMed (MEDLINE), Embase, and the Cochrane Library in order to identify all published articles on dose-response evaluation in SIRT. In order to limit the results, all articles that investigated SIRT in combination with other therapy modalities (such as chemotherapy) were excluded.

**Results:**

A total of 3038 records were identified of which 487 were screened based on the full text. Ultimately, 37 studies were included for narrative analysis. Meta-analysis could not be performed due to the large heterogeneity in study and reporting designs. Out of 37 studies, 30 reported a ‘mean dose threshold’ that needs to be achieved in order to expect a response. This threshold appears to be higher for hepatocellular carcinoma (HCC, 100–250 Gy) than for colorectal cancer metastases (CRC, 40–60 Gy). Reported thresholds tend to be lower for resin microspheres than when glass microspheres are used.

**Conclusion:**

Although the existing evidence demonstrates a dose-response relationship in SIRT for both primary liver tumours and liver metastases, many pieces of the puzzle are still missing, hampering the definition of standardized dose thresholds. Nonetheless, most current evidence points towards a target mean dose of 100–250 Gy for HCC and 40–60 Gy for CRC. The field would greatly benefit from a reporting standard and prospective studies designed to elucidate the dose-response relation in different tumour types.

**Supplementary Information:**

The online version contains supplementary material available at 10.1007/s00259-021-05340-0.

## Introduction

Selective internal radiation therapy (SIRT) is a treatment modality for primary and metastatic liver cancer that has been incorporated into clinical practice worldwide for over two decades [1, 2]. During SIRT, microspheres loaded with the beta-emitting isotopes yttrium-90 (^90^Y) or holmium-166 (^166^Ho) are administered through a microcatheter positioned in the hepatic artery. The heterogeneous dose distribution that the microspheres provide to the liver has been under investigation ever since the first implementation of SIRT in clinical studies, typically expressed in a tumour to normal tissue ratio (T/N ratio). In the early days of SIRT, the procedure was performed through laparotomy followed by direct injection of ^90^Y microspheres into the hepatic artery. The dose distribution could then be measured with a beta probe directly at the liver surface, resulting in T/N ratios up to 45:1 [1]. More recently, a second level of dose heterogeneity has become a topic of interest: the heterogeneous dose distribution within a tumour, typically visualized in a dose-volume histogram (DVH) [3, 4]. Finally, the biological effect of SIRT on healthy liver tissue and its impact on toxicity is an important factor. Compared to the dose-response studies in SIRT, relatively few studies have addressed this issue. The dose distribution on healthy tissue in relation to toxicity is however beyond the scope of this review.

Clinical results of SIRT have always been variable between patients, stressing the need for further in-depth dosimetry and dose-response analysis, as the mean tumour dose has often been correlated to both tumour response and survival [5, 6]. Most research has been performed with ^90^Y microspheres (*E*_β-max_ = 2.28 MeV (100%)), of which two (vastly different) commercial products are available: glass microspheres with a high specific activity (up to 5000 Bq per microsphere) and resin microspheres with a lower specific activity (50 Bq per microsphere) [7, 8]. As a direct result of the difference in specific activity, a much lower number of microspheres is administrated during treatment with glass microspheres compared to resin microspheres (1.2 million vs. 40–80 million) [7]. Despite the differences in microsphere distribution that could be expected between the two different yttrium products as a result of the mentioned characteristics, both have been shown to be similarly efficacious [9, 10].

During planning angiography days to weeks prior to treatment, technetium-99m-labelled albumin macroaggregates (^99m^Tc-MAA) are administered. The resulting ^99m^Tc-MAA SPECT imaging has also been used for dosimetry, even though this is a surrogate for the actual distribution of microspheres and the validity of this proxy has been questioned multiple times [11–13]. As ^90^Y decay lacks gamma radiation, nuclear imaging after SIRT was initially restricted to SPECT imaging of bremsstrahlung with poor spatial resolution. ^90^Y decay however also involves positron emission in 0.003% of decays [14], and PET-imaging was adopted as an additional imaging modality in 2010, yielding images with a higher spatial resolution that are more fitting for dosimetry, providing a definitely improved quantification accuracy compared to ^90^Y bremsstrahlung SPECT [15]. Next to the ^90^Y microspheres, ^166^Ho microspheres are the third commercially available product for SIRT [16–18]. ^166^Ho emits a slightly less energetic spectrum of beta radiation (*E*_β-max_ = 1.85 MeV (48.8%), 1.77 MeV (49.9%)) but additionally emits gamma radiation suitable for quantitative SPECT imaging (*E*_γ_ = 81 keV (6.6%)), and holmium makes the microspheres paramagnetic, allowing for MRI-based post-treatment dosimetry at a very high resolution [18–21].

Over the years, multiple clinical studies have been performed in order to grasp the tumour dose-response relation after SIRT for a wide array of primary and metastatic liver malignancies such as hepatocellular carcinoma (HCC) [5], biliary tract cancer (BTC) [22], colorectal cancer (CRC) [6], neuroendocrine tumours (NET) [23], and (ocular) melanoma [24]. Even though the heterogeneity of study designs is rather large, many have described a so-called ‘threshold dose’ that needs to be achieved in order to achieve an objective response or at least local disease control. In 2018, a review article on the physics of SIRT has been published [25], which elaborates on many aspects of SIRT, such as pretreatment dosimetry methods, nuclear imaging strategies, and post-treatment dosimetric models. This systematic review adds to the aforementioned study by aiming to collect all studies that have investigated the tumour dose-response relationship after SIRT and to critically appraise the evidence at hand.

## Methods

### Search strategy

On the 31st of January 2020, an initial search was conducted in the following electronic databases: PubMed (MEDLINE), Embase, and the Cochrane Library. The full search strategy can be found in Supplementary Table 1. In brief, we searched for all synonyms for SIRT, but not the actual acronym SIRT, as that yielded a lot of extra results which were mainly about the sirtuin gene and not about radionuclide therapy. Even though the scope of the review clearly consists of dose-response evaluation, it was decided not to include this in the search strategy, as it is difficult to capture in a proper search term and this strongly limited the number of results. After the full-text screening was completed, a second search was performed on the 3rd of July 2020, to add articles that were published during the screening process. Reference lists of all included studies after full-text screening were used for manual cross-referencing. In between the completion and submission of this review, the results of the DOSISPHERE-01 trial were published [26]. As this is the only level 1 evidence available, it was decided to incorporate this study as well.

### Inclusion criteria

Studies were considered eligible for full-text screening if they presented original research on SIRT of liver malignancies. A second criterion was that the title or abstract had to include at least one of the following terms: overall survival (OS), progression-free survival (PFS), or response criteria (e.g. RECIST, PERCIST, WHO, EASL). If this was not the case but the article was clearly about dosimetry based on the title and abstract, it was also included for full-text screening.

### Exclusion criteria

Articles were excluded for full-text screening if they were not about SIRT, studied an isotope other than ^90^Y or ^166^Ho, or were about technical aspects of the treatment such as imaging technicalities or materials used. Reviews, case reports, comments, editorials, and study protocols were excluded, as well as all preclinical work. To further narrow down the results, all studies were excluded in which the effect of SIRT was investigated in combination with other therapies such as chemotherapy and immunotherapy.

### Full-text screening

The full-text screening consisted of two selection rounds, of which the first was to exclude all articles that did not mention a liver dose or tumour dose at all. In the second round, the remaining full texts were screened more thoroughly on whether dose-response evaluation was performed. Only these articles were included in the final analysis.

All studies were assessed for eligibility independently by two reviewers (JR and NJMK). All full-text screening and data extraction were performed by JR. Disagreements were resolved by consensus or by another reviewer if deemed necessary (JFWN).

### Analysis

Due to the extensive heterogeneity in study designs, study populations, outcome measures, and reporting, it was not deemed possible to perform a meta-analysis. Therefore, a narrative analysis was performed.

### Quality of evidence

When writing a systematic review, an important aspect is evaluating the quality of evidence, preferably applying validated risk of bias tools such as the Grading of Recommendations Assessment, Development, and Evaluation (GRADE) system [27]. However, considering the high variability in study designs and reporting strategies and as the majority of the results are retrospective studies, we saw no possibilities to apply such validated risk of bias tools. An estimation of the overall quality of evidence was made taking the GRADE principles into account which is reported at the end of the results section.

## Results

**Fig. 1:**
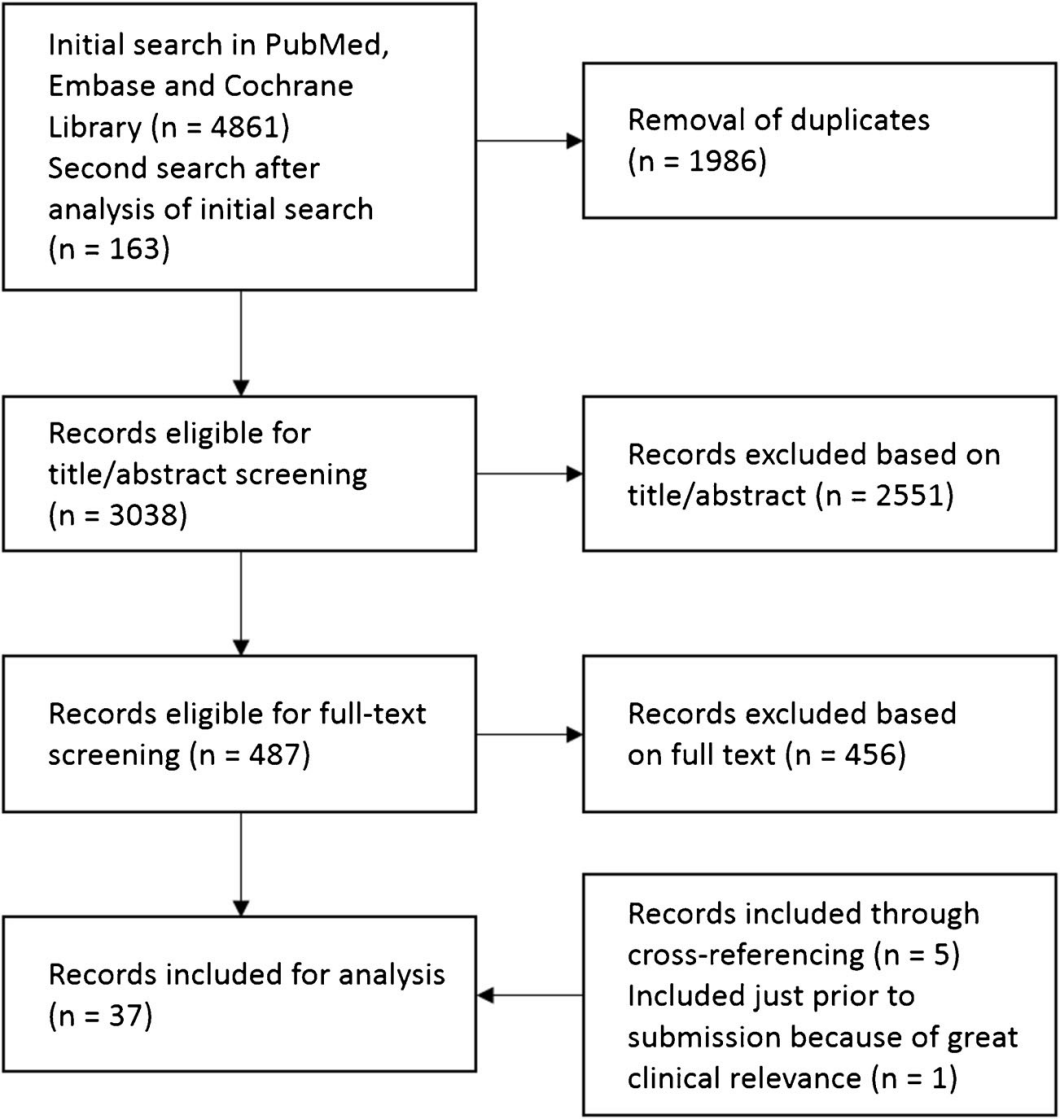
Flow chart of the search strategy and inclusion/exclusion process

The screening process is depicted in Fig. 1. Briefly, our search strategy resulted in 3038 hits after the removal of duplicates, of which 487 publications were considered for full-text screening. Ultimately, 37 articles were included for final analysis. The resulting studies have been subdivided into three groups: studies on HCC patients, studies on non-HCC patients, and studies with mixed patient populations. These studies are summarized in Table 1 (HCC treated with glass microspheres), Table 2 (HCC treated with resin microspheres), Table 3 (non-HCC patients), and Table 4 (mixed populations).

**Table 1:** Included articles on HCC patients treated with glass microspheres

Author	Year	Patients (n)	Lesions (n)	PVT (%)	Criteria	Response rate	Activity prescription method	Dosimetry modality	Dosimetry technique	Dose threshold	Meaning	Effect on median OS
Riaz [30]	2011	84	–	37%*	WHO, EASL	59% (WHO), 81% (EASL)	120 Gy ILD	Tumour hypervascularity ratio	Suborgan (MIRD)	–	No significant difference between responders and non-responders	–
Chiesa^1^ [31]	2011	46	70	–	EASL	38%	120 Gy ILD	^99m^Tc-MAA SPECT	Voxel-based	257 Gy, 400 Gy	Predictor of response, 85% sensitivity, 70% specificity (257 Gy), specificity 86% (400 Gy)	–
Mazzaferro^1^ [35]	2013	52	65	67%	EASL	40%	120 Gy ILD	^99m^Tc-MAA SPECT	Voxel-based	500 Gy	Predictor of response, AUC = 0.78	–
Chiesa^1^ [36]	2015	52	65	67%	EASL	40%	120 Gy ILD	^99m^Tc-MAA SPECT	Voxel-based	250 Gy, 1000 Gy	Tumour control probability ≥50%. Lower dose needed for lesions <10 ml (250 Gy)	–
Garin [32]	2012	36	58	44%	EASL	67%	120 Gy ILD	^99m^Tc-MAA SPECT	Suborgan (MIRD)	205 Gy	Predictor of response, 100% sensitivity, 75% specificity	18 vs. 9 mo
Garin [34]	2013	71	–	45%	EASL	78.8%	One group 120 Gy ILD, other group 205 Gy TD, <120 Gy healthy liver dose	^99m^Tc-MAA SPECT	Suborgan (MIRD)	205 Gy	Predictor of response, 100% sensitivity, 53% specificity	23.2 vs. 11.5 mo
Garin [5]	2015	41	–	100%	EASL	85.4%	205 Gy TD, <120 Gy healthy liver dose	^99m^Tc-MAA SPECT	Suborgan (MIRD)	205 Gy	Predictor of response, 100% sensitivity, 90% accuracy	18.2 vs. 4.3 mo
Garin [37]	2017	85	132	36%	EASL	80.2%	80–150 Gy ILD	^99m^Tc-MAA SPECT	Suborgan (MIRD)	205 Gy	Predictor of response, TCP = 89.7% (>205 Gy) vs. TCP = 9.1% (<205 Gy). Also prolonged OS.	21 vs. 6.5 mo
Garin [26]	2020	58	–	67%	EASL, RECIST 1.1	36–71%	One group 120 Gy ILD, other group 205 Gy TD, <120 Gy healthy liver dose	^99m^Tc-MAA SPECT	Suborgan (MIRD)	205 Gy	Predictor of response, TCP = 71% (>205 Gy) response vs. TCP = 36% (<205 Gy). Also prolonged OS	26.6 vs. 10.7 mo
Kokabi [40]	2014	18	–	100%	NA	NA	120 Gy ILD	^90^Y-SPECT	Suborgan (MIRD)	100 Gy	Predictor of prolonged survival	13.2 vs. 4.3 mo
Srinivas [43]	2014	56	98 (48**)	–	mRECIST	48%	120 Gy ILD	^90^Y-PET	Suborgan (MIRD)	–	No significant difference between responders and non-responders	–
Haste [12]	2017	62	–	–	RECIST, vRECIST, mRECIST	81% (mRECIST)	As recommended by manufacturer (probably 120 Gy ILD)	^99m^Tc-MAA SPECT and ^90^Y-PET	Suborgan (MIRD)	–	No significant difference between responders and non-responders	–
Chan [45]	2018	27	38	52%	mRECIST	84%	At least 120 Gy ILD	^90^Y-PET	Voxel-based	200 Gy	Predictor of response, sensitivity of 66%, specificity of 100%. AUC 0.875.	–
Ho [46]	2018	62	–	30%	MTB decrease ≥50%	59.70%	Calculated based on various parameters, compartment model	^90^Y-PET	Suborgan (MIRD)	152–262 Gy	Predictor of response. ^11^C-acetate avid tumours require a lower dose (152 Gy) than ^18^F-FDG avid tumours (262 Gy). Sensitivity 89.2%, specificity 88%	–
Kappadath [41]	2018	34	53	85%*	WHO, RECIST, mRECIST	56.5% (mRECIST)	120 Gy ILD	^90^Y-SPECT	Voxel-based	160 Gy	TCP_160 Gy_ = 50%	–

Articles are sorted chronologically and are grouped per research group when possible

Dose thresholds indicate mean dose, unless indicated otherwise

All effects on median overall survival are significant unless indicated otherwise, *p* values are not reported

*HCC*, hepatocellular carcinoma; *ILD*, injected liver dose; *MIRD*, medical internal radiation dose; *MTB*, metabolic tumour burden; *n.s.*, not significant; *OS*, overall survival; *PVT*, portal vein thrombosis; *TCP*, tumour control probability; T*D*, tumour dose

*These represent the percentage of BCLC-C class patients when PVT percentages were not provided

**For this lesion-based dose-response analysis, only 48 lesions were included

^1^These articles use the same patient population, Chiesa (2011) contains preliminary results of Mazzaferro (2013)

In general, a large variation in study designs and outcome reporting was found. The included studies applied the following spectrum of response criteria: RECIST (6/37), RECIST 1.1 (8/37), mRECIST (8/37), vRECIST (3/37), EASL (12/37), WHO (5/37), Choi (1/37), EORTC (2/37), and criteria based on metabolic response such as PERCIST or more generally a decrease in metabolic tumour burden (6/37). Additionally, the timing of response evaluation varies greatly, as some studies evaluate the response at set time points such as 1, 3, or 6 months, while others choose the best radiological outcome time point or do not describe the timing of response evaluation at all.

Almost all studies (30/37) have defined a ‘mean dose (*D*_mean_) threshold’ based on their research that should be achieved in order to improve the chance of a beneficial treatment outcome. As described later on, there is no consensus on what the clinical value of this threshold should imply. Most of these studies (28/30) have correlated this threshold to an improved radiological or metabolic response rate, although there is heterogeneity among response assessment methods and reporting of the characteristics of the threshold as well.

### Hepatocellular carcinoma (HCC)

In total, 23 studies have been performed on HCC patients, most of which have used glass microspheres (15/23 studies; 65%). Notably, there is a large variation in the methods used for dosimetry. Two very early studies have determined the T/N ratio through direct, intraoperative measurement of the ^90^Y decay with a beta probe [28, 29]. One study has attempted to make an estimation of the T/N ratio by incorporating the hypervascularization status (based on CT imaging) into a volumetric partition model [30]. A total of 11 studies have predicted the tumour doses based on ^99m^Tc-MAA SPECT [5, 12, 26, 31–38], four have imaged the microsphere distribution with ^90^Y-SPECT [39–42], and six studies have utilized ^90^Y-PET [3, 12, 43–46]. This has also resulted in a very wide range of reported dose thresholds, varying from 61 Gy to 1000 Gy, with the majority being between 100 and 250 Gy (13/19 studies; 68%). Thresholds for glass microspheres are definitely higher than for resin microspheres (range 100–1000 Gy vs. 61–300 Gy).

The first study by Lau et al. from 1994 [28] has set a dose threshold of 120 Gy in order to predict response, although it is not clearly described in the article how this threshold was chosen. In this article, a reference was made towards earlier studies utilizing iodine-131-lipiodol for the treatment of HCC, but the referenced study does not provide further details regarding the 120 Gy cut-off for efficacious treatment either. The only other study applying a similar dosimetric approach [29] has managed to deliver a dose ≥120 Gy to all tumours but has reached a low response rate of only 22.5%.

Only three studies have failed to demonstrate a dose-response relationship. The study by Riaz and colleagues [30] has incorporated a so-called hypervascularity ratio based on angiographic imaging into their dosimetric considerations, a technique that has not been used in later studies. In the discussion of Srinivas et al. [43], it was mentioned that the study is very likely to have been underpowered (*n* = 56 patients). In the third study [12], it was pointed out that their high response rate (81%) and therefore small group of non-responders (*n* = 7) is a possible explanation for the lack of correlation between absorbed dose and radiological response.

As mentioned before, another point of interest is the actual meaning of the defined dose thresholds. For instance, the studies by Garin et al. [5, 26, 32, 34, 37] have all applied the 205-Gy predicted dose threshold that was defined in the first study [32], which was chosen in order to achieve a sensitivity of 100%, or in other words, the author chose to set the response threshold at the minimal dose of responding lesions, corresponding to a tumour control probability (TCP) of 100%. The specificity was 75% in the first study and 53% in the second [34]. Other studies have, for instance, chosen their threshold to obtain a specificity of 100% [45], strived for 50% TCP [41], or have used Youden’s index in order to choose a threshold [44].

Three articles were based on (nearly) the same patient group: the 2011 study of Chiesa and colleagues [31] has presented preliminary data of the publication by Mazzaferro et al. [35], and this patient cohort was re-used for further analysis in 2015 [36]. Interestingly, these studies have proposed vastly different dose thresholds of 257/400 Gy (257 Gy for maximal Youden index, 400 Gy threshold for increased specificity) [31], 500 Gy [35] and 250/1000 Gy (250 Gy for TCP_50_ for lesions smaller than 10 ml, and 1000 Gy for TCP_50_ for lesions threshold for lesions larger than 10 ml) [36]. The last study bases the threshold on the dose that is required in order to achieve a tumour control probability of 50%, in which tumour control is defined as a combination of CR and PR based on EASL criteria.

The study by Ho et al. [46] is the only HCC study that has based response evaluation on nuclear imaging through FDG-PET. In this study, it was described that the dose threshold is dependent on the cellular differentiation state of the tumours, which was assessed through ^11^C-acetate and [^18^F]-FDG PET/CT imaging. ^11^C-acetate avidity was used as a surrogate for a well-differentiated state, whereas [^18^F]-FDG avidity was a surrogate for a poorly differentiated state. The resulting dose thresholds for achieving a metabolic tumour burden reduction of 50% are different, i.e. 152 Gy for ^11^C-acetate avid tumours (sensitivity 90.5%, specificity 87.5%) and 262 Gy for [^18^F]-FDG avid tumours (sensitivity 75.0%, specificity 91.7%).

Two studies are especially noteworthy, of which the first is the recently published ancillary study of the (negative) prospective SARAH trial. In this this study, the relationship of ^99m^Tc-MAA-based, predicted tumour dose to survival and response (RECIST 1.1) was investigated. In the original publication on the SARAH trial [47], no difference in survival was found between SIRT with resin ^90^Y microspheres and sorafenib treatment. However, in this secondary analysis, a clear dose-response relationship has been found, as the highest disease control rate was found in patients of whom the predicted tumour dose exceeded 100 Gy and in whom there was an optimal agreement between ^99m^Tc-MAA SPECT and ^90^Y-SPECT or ^90^Y-PET post-treatment [38]. The second study is the DOSISPHERE-01 trial, which is the first prospective study in nuclear medicine therapy designed to elucidate the benefit of personalized dosimetric treatment planning, in this case, a personalized dose of glass ^90^Y microspheres to patients with HCC. In the intervention group, pretreatment dosimetry was based on tumour uptake on the ^99m^Tc-MAA SPECT, with the goal of achieving a dose >205 Gy to the index lesion. The control group received standard pretreatment dosimetry, i.e. 120 Gy targeted to the perfused liver volume. In this study, it was clearly demonstrated that personalized dosimetric treatment planning resulted in a significant increase in objective response (EASL criteria, 71% vs. 36%) and a survival benefit (26.6 mo vs. 10.7 mo), without an increase in liver toxicity [26].

Eight retrospective studies [5, 28, 32, 34, 37, 38, 40, 42] have also found a significant survival benefit in patients of whom the tumour dose reaches their reported threshold (range 100–205 Gy), most of which are a two to threefold increase in overall survival.

### Non-HCC cancers

Six studies have been performed on hepatic metastases of CRC [4, 6, 48–51], one on BTC [22], one on melanoma [24], and one on NETs [23]. Almost all of the non-HCC studies have been performed with resin microspheres, except for the CRC study using ^166^Ho-PLLA microspheres [51] and one study on BTC with glass microspheres [22].

The reported dose thresholds for CRC are more in line with each other than the thresholds for HCC: the total range over all five yttrium-based studies was 40 to 60 Gy. Three studies have used the same microspheres (resin) and the same response criterion of a total lesion glycolysis (TLG) reduction ≥50% on FDG-PET follow-up and have also used ^90^Y-PET for dosimetry. In the study by Van den Hoven and others [49], the presented *D*_mean_ threshold of 40–60 Gy is a conservative estimate. Herein, it was also demonstrated that the baseline TLG is associated with the extent of metabolic response. Willowson et al. found that a *D*_mean_ greater than 50 Gy predicted a metabolic response with a positive predictive value of 91% [4]. The third study [6] demonstrated that a *D*_mean_ ≥ 60 Gy was a predictor for metabolic response with a specificity of 95% and sensitivity of 70%. Additionally, they have described that a threshold of 39 Gy or lower can be used to predict a non-response with a sensitivity of 80% and specificity of 95%.

Similar to HCC, three studies [4, 48, 49] report that a difference in *D*_mean_ is correlated to a prolonged overall survival for CRC patients, most dramatically a fourfold difference in overall survival in the study by Lam et al. (*D*_mean_ threshold of 55 Gy) [48].

One recent study has explored the dose-response relationship in patients with CRC that have been treated with ^166^Ho-poly(L-lactic acid) (PLLA) microspheres. The threshold of 90 Gy (100% sensitivity/TCP_100_) was, as described by the authors, difficult to compare to the abovementioned thresholds found in yttrium-based studies because of numerous reasons as differences in specific activity and half-life [51].

The study by Eaton et al. [24] on 7 melanoma patients (resin microspheres) has found a correlation between the percentage of a tumour volume that received >50Gy and the extent of [18F]-FDG-PET response (decrease in SUV_max_). The studies on BTC [22] (glass microspheres) and NET [23] (resin microspheres) result in *D*_mean_ thresholds of 260 Gy (sensitivity 73.7%, specificity 80%) and 191.3 Gy (sensitivity 83%, specificity 93%), respectively, which are more in the range of HCC studies. Similar to the publication by Levillain et al. [6], Chansanti and colleagues describe that a *D*_mean_ lower than 72.8 Gy was a predictor of non-response in patients with intrahepatic NETs, with a sensitivity of 100% [23].

### Mixed populations

The study by Song et al. [52] mainly studied HCC patients (69.5%) and BTC patients (13%) treated with resin microspheres, which resulted in a *D*_mean_ threshold of 200 Gy (seemingly arbitrarily chosen, a predictor for prolonged PFS of 286 vs. 92 days, i.e. 9.4 vs. 3.0 mo). This is in line with the results on HCC and BTC described above, in which these tumour types appear to require a higher dose than, for instance, CRC. Fowler and colleagues [53] have found a significant dose-response relationship only for CRC (resin microspheres), with a reported dose threshold of 29.8 Gy (sensitivity 76.9%, specificity 75.9%). It has to be noted that all patient subgroups in this study were relatively small (<10 patients per group). In the article published by Lam et al., [54] a relatively large patient cohort (*n* = 122) treated with either glass or resin microspheres was studied, resulting in an independent association between predicted dose (based on ^99m^Tc-MAA) and survival, after stratifying for tumour type (univariate and multivariate analysis). No mean dose thresholds were identified. Only one other study (with either glass or resin microspheres) performed on a mixed population looked into survival as well, in which a difference in overall survival was found between patients of whom the mean tumour doses exceeded 280 Gy and patients with lower tumour doses (TCP_95_, OS 17.7 mo vs. 9 mo) [55].

One publication using ^166^Ho microspheres has investigated the dose-response relationship after SIRT in a mixed population [56]. This study has linked the (geometric) mean tumour dose to both local response as well as survival (linear mixed-response model, log-rank test), similar to all work that has been performed with ^90^Y microspheres. No dose threshold was determined.

### Tumour dose heterogeneity

One of the earliest studies reporting the heterogeneity of the dose distribution within a tumour was performed by Kao et al. in 2013 (resin microspheres) [3], in which it was decided to report the *D*_70_ (minimum dose delivered to 70% of the tumour) and *V*_100_ (percentage of tumour volume receiving ≥100 Gy) values based on the acquired DVHs. A *D*_70_ > 100 Gy was (arbitrarily) suggested as a threshold to predict treatment response in HCC. In the study on HCC patients by Kappadath et al. (glass microspheres), *D*_mean_ and *D*_20_ to *D*_80_ were found to be correlated to mRECIST response, but not *D*_10_, *D*_90_, and *D*_100_ [41]. Willowson and colleagues investigated dose-response in CRC patients [4] and found that the use of *D*_70_ as a dose metric may be favourable to *D*_mean_ as this resulted in a stronger correlation between the dose metric and the outcome, albeit very subtle. Moreover, it is suggested to incorporate a measure of tumour dose heterogeneity such as the coefficient of variance into the dose-response analysis, as this improved the positive predictive value of the prediction model. Last, a study on a relatively small, mixed cohort indicated that DVH-derived dose metrics such as *D*_70_ are more important for predicting response in hypovascularized lesions than in hypervascularized lesions [53].

### Quality of evidence

It is difficult to correctly apply the GRADE approach [27] to this systematic review, as the scope of the review is not to study the extent of clinical benefit of SIRT directly. The objective of elucidating the dose-response correlation would, for instance, not benefit from a control group incorporated in the study design. However, we can state that almost all included studies have been retrospectively executed. For some studies, it is unclear whether patient cases have been re-used for later publications by the same authors, which is a direct disadvantage of the retrospective study designs. If the quality of this body of evidence were to be rated through the GRADE approach, the level would be low.

The most important GRADE category in this body of evidence is the inconsistency between studies, on many levels of study design: patient populations, response criteria, time to follow-up, and applied dosimetry techniques. However, despite this large heterogeneity in study approaches, the relation between mean tumour dose and response or prolonged overall survival has been demonstrated in over 20 studies. We therefore consider it rather likely that the effect of absorbed tumour dose on response and even survival is genuine, albeit unclear what the true mean tumour dose is that needs to be achieved in order to expect a response after SIRT.

## Discussion

In this systematic review, all available evidence on the extent of the tumour dose-response relation in SIRT has been summarized. In the past 25 years, many research groups have investigated this correlation, leading to a broad scale of results. The main finding of this systematic review is that there clearly is a dose-response relation in SIRT (as demonstrated in 34/37 included studies), although it remains difficult to thoroughly characterize this relation, particularly in terms of biological effectiveness. The two most frequently studied cancer types are HCC and CRC and the recommended *D*_mean_ for HCC (100–250 Gy) appears to be higher than for CRC (40–60 Gy). Reported thresholds are lower for resin microspheres than when glass microspheres are used (for HCC: range 61–300 Gy vs. 100–1000 Gy).

A second major finding is the lack of a designing and reporting standard between the various studies. Some heterogeneity between different studies speaks for itself, such as the choice for specific response criteria for specific tumour types (e.g. mRECIST for HCC and RECIST 1.1 for CRC), but the heterogeneity in response criteria is much more extensive than that. Other aspects concerning the choice and implementation of response criteria should be noted as well. It has, for instance, been demonstrated that a metabolic response is achieved earlier after SIRT than anatomic response (1 mo vs. 3 mo) [57]. There is however no clear consensus between different studies on the optimal timing of the response evaluation: timing varies between 1, 3, and/or 6 months after treatment, and some studies reported the best response found over multiple evaluation time points. Moreover, some studies strive for disease control, others for an objective decline in tumour volume (i.e. partial response and complete response).

A similar problem arises when comparing the reported dose thresholds, as there is a wide variety of methods through which thresholds were chosen. There is a large variation as it is not clearly defined whether to adhere, for instance, 100% sensitivity or specificity or a completely different outcome prediction parameter. As a TCP curve is a sigmoid and not a step function, another variance is the fact that not all studies express the same TCP that their threshold results in. Such fundamental differences in study designs and reporting make it impossible to combine data from different studies.

Post-treatment dosimetry is a crucial step in establishing the dose-response relation after SIRT. Obviously, the applied dosimetry techniques have improved and therefore changed over the 25 years of research incorporated in this systematic review. The downside is that this is another aspect that impedes the comparison of the included studies. Of the 37 incorporated articles, 17 have not evaluated the dose post-treatment but have estimated the absorbed dose based on ^99m^Tc-MAA SPECT imaging prior to treatment. Even though the validity of this approach has been a point of discussion [11–13], the studies by Garin and colleagues [5, 26, 32, 34, 37, 58] have, for instance, demonstrated its clear value in predicting the outcome of HCC patients. Optimization through pretreatment dosimetry (in which activity prescription is based on imaging before treatment) is vital for improving the patient outcome, but only post-treatment dosimetry provides information on the technical success of the treatment and the actual dose distribution. Even though pretreatment dosimetry is helpful in predicting response and could lead to its own tumour-response prediction model, a thorough understanding of the true dose-response relation is still lacking, and that can only be evaluated through post-treatment dosimetry. We therefore argue the added value of basing the evaluation of dose-response in SIRT on ^90^Y-PET-based and ^166^Ho-MRI-based dosimetry. These imaging modalities do directly visualize the achieved dose distribution in a high-resolution and are therefore most fit for elucidating the dose-response relation.

In order to truly unravel the dose-response characteristics after SIRT, we require, for each histology and kind of microsphere, a universally adopted, standardized pretreatment and post-treatment dosimetry protocol, as well as a reliable radiological response assessment method, and a well-defined methodology to fix an efficacy threshold. This is also illustrated by the ancillary study of the SARAH trial [38], in which dosimetry was performed based on pretreatment ^99m^Tc-MAA SPECT imaging, because post-treatment imaging was often lacking and there was no consensus on performing ^90^Y-based PET or SPECT imaging between a large number of cooperating centres. Large prospective clinical trials with dosimetry and response evaluation as a primary end point, such as the recently published DOSISPHERE-01 trial [26], are naturally more valuable in resolving this matter than retrospectively executed studies, even though a meta-analysis of (a selection of) the presented retrospective studies in which the data would be re-evaluated in a standardized manner could potentially also provide a large step in the right direction.

The systematic review has demonstrated that resin microspheres seem to result in a different dose threshold than glass microspheres. This may partially be explained by the heterogeneity of the distribution of microspheres as a direct result of the injected number of microspheres and therefore the heterogeneity of the resulting dose distribution, which can be analysed through simulations of DVH’s and DVH-derived dose metrics at a microscopic scale [59]. Several studies have incorporated measures such as *D*_70_ as opposed to the *D*_mean_ that results from MIRD-based suborgan dosimetry. It has been suggested that DVH-derived dose metrics are more important in predicting the outcome of hypovascularized lesions such as CRC metastases [53]. One can imagine that the highly vascularized status of lesions such as HCC is intrinsically resulting in a more homogeneous microsphere distribution in which the microspheres are positioned closer to each other, simply as a result of the higher density of arterioles in which the microspheres can lodge. In a simulation study by Pasciak et al., it has been demonstrated that a decrease in the number of microspheres will lead to a decrease in *D*_70_ and will decrease the steepness of the slope of the DVH [60]. This effect can be compensated for by increasing the total administered dose, i.e. the specific activity of the microspheres, which is the exact difference between the glass and resin ^90^Y microspheres. This may partially explain the differences in *D*_mean_ thresholds that are found, in which glass microsphere treatments appear to require a higher *D*_mean_ than resin microspheres.

## Conclusion

Even though there is extensive evidence for a dose-response relationship in SIRT for both primary liver tumours and liver metastases, many pieces of the puzzle are still missing. This review indicates that the mean absorbed dose threshold to expect a response appears to be higher for HCC (100–250 Gy) than for CRC (40–60 Gy) and also depends on the type of microsphere used (for HCC: range 61–300 Gy vs. 100–1000 Gy). Other than two prospective, randomized trials (DOSISPHERE-01 and SARAH), of which only the first had dosimetry as a primary focus, the quality of evidence is low, precluding any definitive conclusions. Therefore, the field would greatly benefit from a reporting standard and prospective studies designed to further elucidate the dose-response relation in different tumour types. In the past 2 years, two expert panels have formulated recommendations for personalized dosimetry for glass microspheres for HCC treatment [61] and resin microspheres for HCC and other cancer types [62]. We argue that any prospective trial into the effectiveness of SIRT should incorporate standardized dosimetry, in order to at least evaluate the technical success of the treatments performed. In our opinion, this standardization should at least include voxel-based post-treatment dosimetry with high-resolution imaging, resulting in not only a *D*_mean_ but also a measure of dose heterogeneity within the tumours. Secondly, the field would benefit from a standardized response evaluation method and standardized methods through which dose thresholds are defined (such as a TCP of 50%). We expect an instance such as the EANM to formulate guidelines to facilitate and streamline this process in the coming years (Tables 2, 3, 4).

**Table 2:** Included articles on HCC patients treated with resin microspheres

Author	Year	Patients (n)	Lesions (n)	PVT (%)	Criteria	Response rate	Activity prescription method	Dosimetry modality	Dosimetry technique	Dose threshold	Meaning	Effect on median OS
Lau [28]	1994	16	–	–	≥50% decrease in tumour volume	50%	Not clearly described	Beta probe	Suborgan (MIRD)	120 Gy	Predictor of response (87.5% vs. 12.5% response rate)	12.9 vs. 6.0 mo
Ho [29]	1997	71	–	–	≥50% decrease in tumour volume	22.5%	Not clearly described	Beta probe	Suborgan (MIRD)	225 Gy (for response)	TCP_>225 Gy_ = 37.5% TCP_<225 Gy_ = 10.3%	11.0 vs. 6.8 mo (300 Gy cut-off, n.s.)
Strigari [39]	2010	73	–	2.7%*	RECIST, EASL	54% (RECIST, 77% (EASL)	BSA method	^90^Y-SPECT	Voxel-based	110–120 Gy	Predictor of response in at least 50% of patients	–
Kao [33]	2012	8	–	50%	RECIST 1.1, EASL, WHO	37.50%	Partition model	^99m^Tc-MAA SPECT	Suborgan (MIRD)	91 Gy	Median decrease of index tumour of 58%	–
Kao [3]	2013	7	–	29%	RECIST	63.6%	Not clearly described	^90^Y-PET	Voxel-based	*D*_70_ ≥ 100 Gy	Predictor for complete response	–
Allimant [44]	2018	38	–	80%	mRECIST	31%	BSA method	^90^Y-PET	Voxel-based	61 Gy**	Predictor of response, 76.5% sensitivity, 75% specificity	–
Tabone [42]	2020	24	–	100%	mRECIST, EASL	54%***	Partition model, <40 Gy to healthy tissue	^90^Y-SPECT	Suborgan (MIRD)	–	Responders received a higher median dose (248 vs. 138 Gy)	30 vs. 11 mo
Hermann [38]	2020	109	–	61%*	RECIST 1.1	20.5%	Modified BSA method	^99m^Tc-MAA SPECT	Voxel-based	100–150 Gy	TCP_100 Gy_ = 72%, TCP_150 Gy_ = 92%	14.1 vs. 6.8 mo

Dose thresholds indicate mean dose, unless indicated otherwise

*BSA*, body surface area; *D*_*70*_, minimum dose that 70% of the voxels received; *HCC*, hepatocellular carcinoma; *MIRD*, medical internal radiation dose; *n.s.*, not significant; *OS*, overall survival; *PVT*, portal vein thrombosis; *TCP*, tumour control probability

*These represent percentages of BCLC-C class patients when PVT percentages were not provided

**This study reported the area under the DVH instead of mean dose, which is equal to the mean dose [63]

***This group also includes stable disease

**Table 3:** Included articles on non-HCC patients

Author	Year	Cancer type	Micro-spheres	Patients (n)	Lesions (n)	Criteria	Response rate	Activity prescription method	Dosimetry modality	Dosimetry technique	Dose threshold	Meaning	Effect on median OS
Lam [48]	2013	CRC	Resin	25	–	RECIST 1.1	26.7%	BSA method	^99m^Tc-MAA and ^99m^Tc- SC SPECT	Suborgan (MIRD)	55 Gy	Predictor of prolonged survival	32.8 mo vs. 7.2 mo
Van den Hoven [49]	2016	CRC	Resin	30	133	PET RECIST, ≥50% TLG reduction	46%	BSA method	^90^Y-PET	Suborgan (MIRD)	40–60 Gy	Predictor of metabolic response (conservative estimation)	11.5 mo vs. 5.3 mo (60 Gy cut-off)
Willowson [4]	2017	CRC	Resin	22	63	TLG ≥50% reduction	67%	Modified BSA method	^90^Y-PET	Voxel-based	50 Gy	Predictor of metabolic response, 91% PPV	–
Levillain [6]	2018	CRC	Resin	24	57	Adapted from PERCIST, ≥50% TLG reduction	64.9%	Partition model	^90^Y-PET	Voxel-based	39 Gy, 60 Gy	Predictor of metabolic response, 39 Gy for <15% TLG reduction, 80% sensitivity, 95% specificity. 60 Gy for ≥50% TLG reduction, 70% sensitivity, 95% specificity	13 vs. 5 mo (39 Gy cut-off)
Abbott [50]	2020	CRC	Resin	23	96	vRECIST	14%	Modified BSA method	^90^Y-SPECT	Voxel-based	48.3 Gy	Predictor of response, odds ratio 1.09	–
Van Roekel [51]	2020	CRC	Ho-166 PLLA	40	–	PERCIST, RECIST 1.1	36% (PERCIST)	60 Gy ILD	^166^Ho-SPECT	Suborgan (MIRD)	90 Gy	Predictor of response, sensitivity 100%, specificity 38%	Improved survival
Bourien [22]	2019	BTC	Glass	64	–	RECIST1.1, Choi	15% (RECIST), 71% (Choi)	80–150 Gy ILD (or higher if segmentectomy)	^99m^Tc-MAA SPECT	Suborgan (MIRD)	260 Gy	Predictor of response (Choi criteria). No correlation RECIST response	28.2 vs. 11.4 mo
Eaton [24]	2014	Melanoma	Resin	7	30	EORTC	60%	Self-modified BSA method	^90^Y-SPECT	Voxel-based	50 Gy	Predictor of metabolic response	–
Chansanti [23]	2017	NET	Resin	15	55	mRECIST	46.7%	Partition model	^99m^Tc-MAA SPECT	Suborgan (MIRD)	72.8 Gy, 191.3 Gy	Predictor of response (191.3 Gy), 83% sensitivity and 93% specificity. <72.8 Gy predicted no response with 100% sensitivity	–

Dose thresholds indicate mean dose, unless indicated otherwise

*BSA*, body surface area; *BTC*, biliary tract cancer; *CRC*, colorectal cancer; *HCC*, hepatocellular carcinoma; *ILD*, injected liver dose; *MIRD*, medical internal radiation dose; *NET*, neuroendocrine tumour; *OS*, overall survival; *SC*, sulphur colloid; *TLG*, total lesion glycolysis

**Table 4:** Included articles on mixed populations

Author	Year	Cancer type	Microspheres	Patients (n)	Lesions (n)	Criteria	Activity prescription method	Response rate	Dosimetry modality	Dosimetry technique	Dose threshold	Meaning	Effect on median OS
Demirelli [55]	2015	12 HCC, 5 BTC, 18 CRC, 6 breast, 11 others	46/54 resin, 8/54 glass	54	–	RECIST, EORTC	BSA method (resin), 100–110 Gy ILD (glass)	33% (RECIST), 48% (EORTC)	^99m^Tc-MAA SPECT	Suborgan (MIRD)	280 Gy	Predictor for tumour control (95% TCP, both criteria), AUC = 0.869	17.7 vs. 9 mo
Song [52]	2015	16 HCC, 3 BTC, 4 others	Resin	23	–	RECIST1.1	Partition model, BSA method	Not clearly reported	^99m^Tc-MAA SPECT, ^90^Y-PET	Suborgan (MIRD)	200 Gy (^90^Y-PET)	Predictor for prolonged PFS. Could not be found for ^99m^Tc-MAA SPECT based dosimetry	–
Lam [54]	2015	26 HCC, 18 BTC, 20 NET, 29 CRC	98/122 resin, 24/122 glass	122	–	mRECIST	Modified BSA method (resin), 90–120 Gy ILD (glass)	48%	^99m^Tc-MAA and ^99m^Tc- SC SPECT	Suborgan (MIRD)	–	Tumour dose independently associated with survival, stratified for tumour type	–
Fowler [53]	2016	8 HCC, 4 NET, 9 CRC, 3 others	Mainly glass for HCC (6/8), mainly resin for other (14/16)	24	92	RECIST, vRECIST	Modified BSA (resin), as manufacturer recommends (glass)	41.3% (RECIST), 73.5% (vRECIST)	^90^Y-PET	Voxel-based	29.8 Gy (CRC)	Predictor of response, 76.9% sensitivity, 75.9% specificity. No significant trend for other tumour types	–
Bastiaannet [56]	2019	21 CRC, 4 breast, 4 cholangiocarcinoma, 4 melanoma, 3 others	Ho-166 PLLA	36	98	PERCIST	60 Gy ILD, 80 Gy ILD (3 patients)	50%	^166^Ho-SPECT	Suborgan (MIRD)	–	Responders had received a higher tumour dose than non-responders. Median OS was higher in responders	–

Dose thresholds indicate mean dose, unless indicated otherwise

*BSA*, body surface area; *BTC*, biliary tract cancer; *CRC*, colorectal cancer; *HCC*, hepatocellular carcinoma; *ILD*, injected liver dose; *MIRD*, medical internal radiation dose; *NET*, neuroendocrine tumour; *OS*, overall survival; *SC*, sulphur colloid

## Supplementary information

ESM 1 (DOCX 13 kb)

## Data Availability

The datasets generated during and/or analysed during the current study are available from the corresponding author on reasonable request.
