# Supplementary material for: To 1000 Gy and back again: a systematic review on dose-response evaluation in selective internal radiation therapy for primary and secondary liver cancer
Source: Eur J Nucl Med Mol Imaging. 2021 Apr 10;48(12):3776–90. doi: 10.1007/s00259-021-05340-0 (PMC8484215; doi:10.1007/s00259-021-05340-0)
Supplement: Supplementary file 1 — (DOCX 13 kb) [file 259_2021_5340_MOESM1_ESM.docx]

**Supplementary data**

Supplementary table 1: Detailed search strategy in the electronic databases.

| Pubmed | |
| --- | --- |
| #1 | selective internal radiotherapy[tiab] OR selective internal radiation therapy[tiab] OR selective intra-arterial radiotherapy[tiab] OR selective intraarterial radiotherapy[tiab] OR selective intra-arterial radiation therapy[tiab] OR selective intraarterial radiation therapy[tiab] OR radio-embolization[tiab] OR radio-embolisation[tiab] OR radioembolization[tiab] OR radioembolisation[tiab] OR TARE[tiab] OR trans-arterial radio*[tiab] OR transarterial radio*[tiab] |
| Embase | |
| #1 | radioembolization/ |
| #2 | (selective internal radiotherapy OR selective internal radiation therapy OR selective intra-arterial radiotherapy OR selective intraarterial radiotherapy OR selective intra-arterial radiation therapy OR selective intraarterial radiation therapy OR radio-embolization OR radio-embolisation OR radioembolization OR radioembolisation OR TARE OR trans-arterial radio* OR transarterial radio*).ti,ab,kw. |
| #3 | #1 OR #2 |
| #4 | Limit #3 to conference abstract status |
| #5 | #3 not #4 |
| Cochrane library | |
| #1 | ("selective internal radiotherapy" OR "selective internal radiation therapy" OR "selective intra arterial radiotherapy" OR "selective intraarterial radiotherapy" OR "selective intra arterial radiation therapy" OR "selective intraarterial radiation therapy" OR "radio embolization" OR "radio embolisation" OR "radioembolization" OR "radioembolisation" OR "trans arterial radio*" OR "transarterial radio*"):ti,ab,kw |
